# The perceived barriers and facilitators in completing a Master’s degree in Physiotherapy

**DOI:** 10.4102/sajp.v74i1.445

**Published:** 2018-05-30

**Authors:** Nicolette Comley-White, Joanne Potterton

**Affiliations:** 1 Department of Physiotherapy, University of the Witwatersrand, South Africa

## Abstract

**Background:**

Participating in postgraduate study is daunting and as yet there is a dearth of literature on what students’ experiences are when obtaining their Master’s degree in Physiotherapy.

**Objectives:**

The aim of this study was to gain insight into the perceived barriers and facilitators in completing a Master’s degree in Physiotherapy.

**Method:**

Semi-structured, in-depth interviews were conducted with 10 physiotherapists who had completed a Master’s degree in Physiotherapy from a university in South Africa, representative of coursework and dissertation Master’s degrees, completed within the stipulated time period as well as taking longer to complete the degree. The topics covered a range of speciality areas. The interviews were transcribed, sent for member checking and analysed thematically.

**Results:**

Within 10 interviews data saturation was reached. Two themes were identified: research environment and support, both of which were seen as either a facilitator or a barrier, depending on the participant. The theme of research environment was divided into categories of workplace and data collection. The second theme, support, was also seen as either a barrier or a facilitator. This theme encapsulated the categories of supervisor support, workplace support and a personal support network.

**Conclusion:**

The research environment and support are two major factors that can influence the experience of obtaining a master’s degree in physiotherapy, both positively and negatively.

**Clinical implications:**

With increasing numbers of physiotherapists obtaining postgraduate degrees, universities need to facilitate the process of obtaining the degree, which will ensure more physiotherapists with postgraduate degrees, thereby strengthening the profession.

## Introduction

Universities around the world are being challenged to increase their numbers of postgraduate students (McCallin & Nayar 2012). This drive to become research intensive has impacted South African universities as well. As a result, questions of student throughput and completion rates have become increasingly relevant in all areas of postgraduate study including physiotherapy (Cobbing et al. 2017). Within the field of physiotherapy, there is a growing interest in specialisation and life-long learning. The above factors, together with the drive to remain internationally mobile, mean that interest in postgraduate degrees such as the Master’s degree in physiotherapy has grown rapidly over the past decade (Glover et al. 2008).

Traditionally in South African universities a Master of Science in Physiotherapy is a postgraduate degree which can be completed by dissertation or coursework with a research report. At the site of this study, the Faculty of Health Sciences has made the recommended time to complete such a degree 2 years for part-time students, with the recognition that many students will require an additional year. This time frame is referred to as ‘n + 1’. Timely completion of research is a problem that has been identified around the world and across many academic fields (Wingfield 2011).

As the pressure to produce more postgraduate students has increased, the number of postgraduate students has risen but the number of research supervisors has remained the same. This pressure has been described around the world (McCallin & Nayar 2012) and is certainly echoed in South Africa (Cobbing et al. 2017).

Participating in postgraduate studies is no small undertaking. While many postgraduate physiotherapy students describe their studies as being very stressful, they also acknowledge that the process was life-changing and had a positive impact on many facets of their lives, both professionally and personally (Stathopoulos & Harrison 2003). In general, some of the barriers that have been identified in research in the field of rehabilitation can be contributed to a lack of funding, research mentors, time, statistical support and familiarity with the research process (Carter & Lubinsky 2016).

There is a growing body of research on the experiences of postgraduate supervision (Bitzer & Albertyn 2011; Drennan & Clarke 2009; Sidhu et al. 2013, 2014) and the barriers and facilitators to *undertaking* a postgraduate degree in physiotherapy (Cobbing et al. 2017; Sran & Murphy 2009). However, there is a striking paucity of research on the barriers and facilitators experienced by students during a master’s degree in physiotherapy.

Thus, the purpose of this study was to add the students’ voices to the multifaceted research experience by determining the perceived barriers and facilitators in completing a Master’s degree in Physiotherapy. Further objectives of the study were to establish the perceived barriers and facilitators in completing a Master’s degree in Physiotherapy for students who completed the degree in ‘n + 1’ or less time, and in those who completed it in over ‘n + 1’ time.

## Methods

Recent graduates with a postgraduate degree of Master’s in Physiotherapy from a research-intensive university in South Africa were invited to participate in the study. Through purposive sampling we ensured that students of both coursework and dissertation (all on a part-time basis), students from a range of physiotherapy speciality areas and students who had completed their degrees both within ‘n + 1’ years and over ‘n + 1’ years were included. Over the past 5 years, the Physiotherapy Department of the University of the Witwatersrand has graduated an average of 11 master’s students per year. Using this population, interview sessions were set up initially with 10 participants to commence data collection and analysis. Data collection continued until data saturation was reached.

### Data collection and analysis

Participants had individual, in-depth semi-structured interviews with the first author (Gill et al. 2008), which explored the participants’ experience of doing their postgraduate master’s degree and what they felt were barriers to and/or facilitators on the journey. The first author asked the questions in an open and friendly manner, allowing the participants to express their own opinions and views. She had not supervised any of the participants during their research. The questions were broad and open-ended and the interview process was fluid. Probing questions were used to draw out details of each participant’s experiences, based on an interview schedule, and the interviews were audio-recorded.

The interviews were transcribed, anonymised (by removing all identifiers such as names, workplaces and supervisors) and then sent for member checking. Substantive statements were identified and inductive analysis was performed (Thomas 2006) to identify themes, categories and sub-categories. Manifest analysis of the data was performed (Bengtsson 2016). Data analysis was performed by both authors separately, who then collaborated to obtain consensus on the sub-categories, categories and themes (Graneheim & Lundman 2004).

### Ethical considerations

Written informed consent was given by the participants for the interviews and the audio-recording thereof. Ethical approval was obtained from the Human Research Ethics Committee of the University of the Witwatersrand (No. M160745).

## Results

Data saturation was reached within 10 interviews. The range of Master’s degrees and time taken to complete the degree are shown in Table 1.

**TABLE 1:** Description of the participants.

Participant	Degree format	Area of physiotherapy	Duration of degree
1	Dissertation	Women’s health	Over n + 1†
2	Dissertation	Orthopaedics	n + 1
3	Dissertation	Cardiovascular	Over n + 1
4	Dissertation	Orthopaedics	Over n + 1
5	Research report	Adult neurology	Over n + 1
6	Research report	Paediatrics	n + 1
7	Research Report	Neuromuscular skeletal	n + 1
8	Dissertation	Paediatrics	Over n + 1
9	Research report	Cardiovascular	n + 1
10	Research report	Paediatrics	Over n + 1

† , ‘n + 1’: This is the time taken to complete the degree, usually 2 years plus the allowance of one extra year.

The participants were split equally between research reports and dissertations, with 6 out of the 10 participants taking longer than n + 1. There was a trend that dissertation degrees took longer than the stipulated time period for completion.

The two themes that were identified were research environment and support. Depending on the participants, these were seen either as a barrier or as a facilitator. The themes, categories and sub-categories are shown in Table 2.

**TABLE 2:** Themes, categories and sub-categories identified in the study.

Theme	Category	Sub-category	
		Facilitator	Barrier
Research environment†	Workplace	Structured time for research	Not supportive of postgraduate studies
		Study leave for degree purposes	
	Data collection	Efficient logistics (methodology)	Subject recruitment
			Subject follow-up
Support	Supervisor	Prompt feedback	Poor communication
		Supervisor expertise	Lack of input
		Supportive and professional	
	Workplace	Employer and/or colleagues supportive of degree	Loss of annual leave and income for data collection and/or writing time
	Personal	Self-motivation	Stress and other life events
		No other responsibilities	
		Strong personal support structures	

† , Research environment refers to the site where the students collected data, not the academic institution where students were registered.

Key quotes from the participants are presented in Table 3.

**TABLE 3:** Participant quotes regarding perceived barriers and facilitators within the themes.

Theme	Barriers	Facilitators
Research environment	‘Data collection for me was very difficult. Getting patients who only had [*specific condition*] was really difficult.’ (Participant 8)	‘I was given study leave so that was very helpful.’ (Participant 1)
	‘The length of the study was quite daunting when I eventually realised how long it was going to take. It took me a very long time to find the 90 subjects.’ (Participant 3)	‘I did my data collection during work hours, because it was basically the patients that I was treating in the ward.’ (Participant 2)
	‘It wasn’t encouraged for us to do any research during working hours, which I didn’t feel right about because we are a massive academic hospital.’ (Participant 6)	‘I think logistics are so important, so the good thing about my research was that I could totally depend on myself.’ (Participant 2)
Support	‘I think I realised for me that I am not a researcher which was quite a hard realisation.’ (Participant 10)	‘So having supportive employers was definitely a help.’ (Participant 5)
	‘It was stressful. I remember being stressed all the time.’ (Participant 2)	‘…family support was amazing… without that support it would be very difficult.’ (Participant 8)
	‘…you don’t want to bother your supervisors because they very much an authority figure [*regarding not receiving sufficient feedback*].’ (Participant 7)	‘I was young and single and living at home with my parents and had no real responsibilities. …When I got home I could sit in my room and just do my work.’ (Participant 2)
		‘I had a wonderful supervisor. [*Name*] was my supervisor and she had personal knowledge on [*the study topic*] and had done a study in the same clinic before; so I think it was wonderful to have her as my supervisor.’ (Participant 3)
		‘And it was nice to have the two [*supervisors*] because I felt like they had different strengths and different weaknesses.’ (Participant 8)

A noteworthy finding was that of the participants who completed the degree within the ‘n + 1’ time period, there was a stronger identification of facilitators than barriers, with personal motivation and efficient logistics (e.g. participant recruitment and follow-up) being the predominant factors. Conversely, those who took longer than the stipulated time period to complete their degree identified more barriers than facilitators, with challenging data collection and poor supervisor relationships dominating.

## Discussion

The two emergent themes, research environment and support, are key factors that influence students’ experience of completing their postgraduate studies, and potentially the time they take to do it. The research environment (which refers to the site where the students collected data, not the academic institution where they were registered as students) needs to be supportive of postgraduate studies, allowing time for data collection, studies and write-up of the dissertation or research report. Recommendations have been made for academic institutions and the Department of Health to implement study leave, allowing for a workplace that is more conducive to research (Cobbing et al. 2017). Furthermore, developing a methodology that allows for uncomplicated and efficient data collection (both participant recruitment and follow-up) is seen as a facilitator of the postgraduate process. Postgraduate students are often unfamiliar with the exact requirements of what research entails, and experience a shift in responsibility when progressing from the structure of coursework to the independent work required for conducting research (Sayed et al. 1998).

With regard to support, supervisor support came across as one of the strongest categories, with key aspects being prompt feedback, supervisor expertise and a professional and supportive supervisor. Our findings are comparable with global literature showing the role of the supervisor in expediting postgraduate studies in other academic fields (Lee 2007; McCallin & Nayar 2012). Furthermore, a major determinant of student success has been identified as supervisor–student relationship (McCallin & Nayar 2012; Sayed et al. 1998), with students claiming that inadequate supervision was the major problem with timely completion of their postgraduate degree (Emilsson & Johnsson 2007). As was found in this study, positive relationships foster success while poor relationships negatively affect timely completion (De Valero 2001; Gurr 2001). Our findings add that a poor relationship may arise because of a lack of supervisor input and/or poor communication from the supervisor. In contrast, students who had a positive relationship with their supervisors identified this as an important facilitator in the research process. With the advent of increased communication via email, and conversely a decrease in face-to-face time between supervisor and student, it is important to work on good communication skills, allowing for a stronger relationship to be built. This is especially relevant when working with long-distance students or students whose workplace does not allow for sufficient time off for regular face-to-face supervisor meetings. Continuing professional development for supervisors is needed to ensure that they have the skills to support their students on their research journey (Lee 2007).

Support within the workplace was another key category that arose from data analysis. This links closely with the theme of research environment and the ethos of the employer or employing institute in providing adequate time and support for data collection, study leave and write-up (Sayed et al. 1998). As the preference of most postgraduate students is to complete their degree on a part-time basis (Sayed et al. 1998; Sran & Murphy 2009), one needs to consider the many other areas of life that a student juggles (Sayed et al. 1998). In addition, potential loss of income (because of lack of workplace support) during the postgraduate degree adds to the stress that a student may already be under. Trivedil, Sheth and Vyas (2013) conducted a study of 100 postgraduate physiotherapy students about the prevalence and source of stress and found that up to 45% of their study participants were stressed and 32% had severe problems and psychological distress, with supervisor relationship, challenges with colleagues and personal life demands all playing a role in their stress levels. These findings speak across the themes and categories identified in our study.

Lastly, personal support was a category to note. The importance of having personal motivation and no adverse life events occurring during the course of a postgraduate degree is highlighted. Life events and the support structure available are seen as important factors in a student’s experience (Sayed et al. 1998) and this links with the sub-category of a supportive and professional supervisor. As Wingfield (2011) succinctly reflects, ‘life happens during postgraduate studies’ and the role of having personal support during such times cannot be underestimated when reflecting on the experiences of students.

### Limitations and recommendations

One of the limitations of this study is that it reflects the experiences of students at only one site. Furthermore, all of the participants were part-time students who were working in a clinical setting at the time of data collection. Full-time students, students from different universities and/or students working in an academic setting may express different experiences.

Based on the experiences expressed by the participants of this study, the authors recommend the following: as a postgraduate supervisor one needs to be mindful of the importance of the relationship and communication methods with the students and provide an adequate level of support; supervisors can help in directing students in their methodology to ensure efficient and realistic logistics. This is also relevant for postgraduate students (current and future) who are planning their studies so that they understand what commitment will be required of them for the duration of their studies. Lastly, employers of potential postgraduate students need to be cognisant of the structure of their workplace and how this may potentially impact students’ journey through their studies.

## Conclusion

Globally there is a trend for increased interest in pursuing postgraduate degrees in physiotherapy. Postgraduate studies are known to be stressful, yet little research has been conducted to explore the experiences of students engaged in postgraduate research. The results of this qualitative study highlighted the importance of the research environment and support as potential barriers and/or facilitators of completing a master’s in physiotherapy. More attention needs to be paid to these factors by employers, research supervisors and the students themselves in order to optimise the research experience of students and expedite the timely completion of their postgraduate degree.
